# The complete chloroplast genome sequence of *Brachypodium sylvaticum*

**DOI:** 10.1080/23802359.2021.1966337

**Published:** 2021-08-18

**Authors:** Yunqiang Wang, Yi Wang, Xiaolong Yuan, Yunqin Li, Qinghua Wang

**Affiliations:** a Yunnan Branch, Institute of Medicinal Plant, Chinese Academy of Medical Sciences, Yunnan Key Laboratory of Southern Medicinal Resources, Kunming, PR China; b Laboratory of Forest Plant Cultivation and Utilization, Yunnan Academy of Forestry & Grassland Science, Kunming, PR China

**Keywords:** *Brachypodium sylvaticum*, chloroplast, Illumina sequencing, phylogenetic analysis

## Abstract

The first complete chloroplast genome (cpDNA) sequence of *Brachypodium sylvaticum* was determined from Illumina HiSeq pair-end sequencing data in this study. The cpDNA is 136,392 bp in length, contains a large single-copy region (LSC) of 80,854 bp and a small single-copy region (SSC) of 12,765 bp, which were separated by a pair of inverted repeats (IR) regions of 21,383 bp. The genome contains 130 genes, including 84 protein-coding genes, 8 ribosomal RNA genes, and 38 transfer RNA genes. Further phylogenomic analysis showed that *B. sylvaticum* and *B. distachyon* clustered in a unique clade in Brachypodium genus.

*Brachypodium sylvaticum* (Huds.) Beauv. 1812 is a perennial self-compatible bunchgrass originating in Asia, Europe, and North Africa (Wolny et al. 2011). It is an excellent candidate for a model perennial grass because it is self-fertile, small, diploid, easy to grow, and has a small genome (Marchini et al. 2018). It was introduced to USA in the early 1900s (Breda et al. 2012) and quickly grown to an invasion plant community of a thick monocultures (Roy et al. 2011). However, there has been no genomic studies on *B. sylvaticum.*

Herein, we reported and characterized the complete *B. sylvaticum* chloroplast genome. The GenBank accession number is MZ043779. One *B. sylvaticum* individual (specimen number: 2020075) was collected from Heilongtan Kunming, Yunnan Province of China (25°14′13″N, 102°71′17″E). The specimen and DNA were deposited at Yunnan Academy of Forestry Herbarium, Kunming, China (contact person: Wang Yi, email: 22825818@qq.com) under the voucher number Wy608. DNA was extracted from its fresh leaves using DNA Plantzol Reagent (Invitrogen, Carlsbad, CA).

Paired-end reads were sequenced by using Illumina HiSeq system (Illumina, San Diego, CA). In total, about 23.27 million high-quality clean reads were generated with adaptors trimmed. Aligning, assembly, and annotation were conducted by CLC de novo assembler (CLC Bio, Aarhus, Denmark), BLAST, GeSeq (Tillich et al. 2017), and GENEIOUS version 11.0.5 (Biomatters Ltd, Auckland, New Zealand). To confirm the phylogenetic position of *B. sylvaticum*, other 22 species of *Pooideae* subfamily from NCBI were aligned using MAFFT version 7 (Katoh and Standley 2013). The maximum likelihood (ML) bootstrap analysis was conducted using RAxML (Stamatakis 2006); bootstrap probability values were calculated from 1000 replicates. *Dendrocalamus sinicus* (MK962316) and *D. latiflorus* (FJ970916) were served as the out-group.

The complete *B. sylvaticum* chloroplast genome is a circular DNA molecule with the length of 136,392 bp, contains a large single-copy region (LSC) of 80,854 bp and a small single-copy region (SSC) of 12,765 bp, which were separated by a pair of inverted repeats (IR) regions of 21,383 bp. The junction specific primers of LSC, SSC, and IR region were designed according to the silico genome sequence, and the junctions of LSC, SSC, and IR region were sequenced by Sanger DNA sequencing method. The result of Sanger DNA sequencing is same of the silico genome sequence. The overall GC content of the whole genome is 38.5%, and the corresponding values of the LSC, SSC, and IR regions are 36.4%, 32.7%, and 44.0%, respectively. The chloroplast genome contained 130 genes, including 84 protein-coding genes, 8 ribosomal RNA genes, and 38 transfer RNA genes. Phylogenetic analysis showed that *B. sylvaticum* and *B. distachyon* clustered in a unique clade in *Brachypodium* genus (Figure 1). The determination of the complete chloroplast genome sequences provided new molecular data to illuminate the *Pooideae* subfamily evolution.

**Figure 1.**
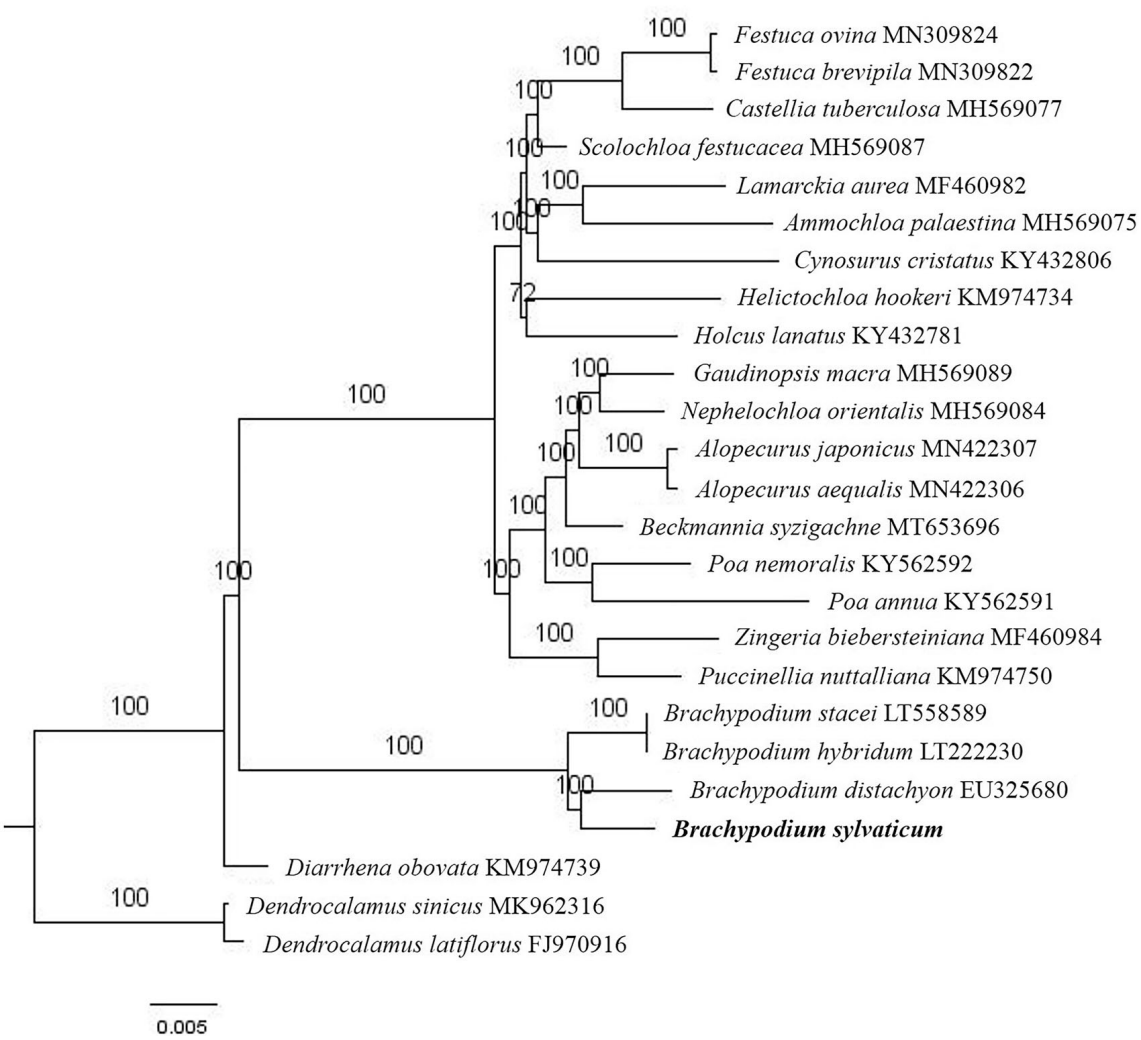
The maximum-likelihood tree based on the 23 chloroplast genomes of subfamily *Pooideae*. The bootstrap value based on 1000 replicates is shown on each node.

## Data Availability

The data that support the findings of this study are openly available in NCBI GenBank database at (https://www.ncbi.nlm.nih.gov) with the accession number is MZ043779, the associated BioProject, SRA, and Bio-Sample numbers of the raw sequence data are PRJNA727716 and SRX10809719, SAMN19030599, respectively, which permits unrestricted use, distribution, and reproduction in any medium, provided the original work is properly cited.
